# A modified surgical technique in the management of eyelid burns: a case series

**DOI:** 10.1186/1752-1947-5-373

**Published:** 2011-08-15

**Authors:** Haiying Liu, Kun Wang, Qigang Wang, Shudong Sun, Youxin Ji

**Affiliations:** 1 Burn Center, Weifang People's Hospital, Weifang Medical School, Weifang, Shandong Province 265000, PR China; 2 Qingdao Central Hospital, Qingdao, Shandong Province 266042, PR China

## Abstract

**Introduction:**

Contractures, ectropion and scarring, the most common sequelae of skin grafts after eyelid burn injuries, can result in corneal exposure, corneal ulceration and even blindness. Split-thickness or full-thickness skin grafts are commonly used for the treatment of acute eyelid burns. Plasma exudation and infection are common early complications of eyelid burns, which decrease the success rate of grafts.

**Case presentation:**

We present the cases of eight patients, two Chinese women and six Chinese men. The first Chinese woman was 36 years old, with 70% body surface area second or third degree flame burn injuries involving her eyelids on both sides. The other Chinese woman was 28 years old, with sulfuric acid burns on her face and third degree burn on her eyelids. The six Chinese men were aged 21, 31, 38, 42, 44, and 55 years, respectively. The 38-year-old patient was transferred from the ER with 80% body surface area second or third degree flame burn injuries and third degree burn injuries to his eyelids. The other five men were all patients with flame burn injuries, with 7% to 10% body surface area third degree burns and eyelids involved. All patients were treated with a modified surgical procedure consisting of separation and loosening of the musculus orbicularis oculi between tarsal plate and septum orbital, followed by grafting a large full-thickness skin graft in three days after burn injury. The use of our modified surgical procedure resulted in 100% successful eyelid grafting on first attempt, and all our patients were in good condition at six-month follow-up.

**Conclusions:**

This new surgical technique is highly successful in treating eyelid burn injuries, especially flame burn injuries of the eyelid.

## Introduction

Eyelid involvement is common in facial burns. Treatment of eyelid burn injuries requires great care for the protection of the cornea. Burns damage tissues primarily by denaturing and coagulating cellular proteins and through vascular ischemic damage [1]. The most common etiologic agents of eyelid burns include local thermal and chemical burns and systemic burns [1,2]. Patients with burns involving the face often also have burns to the eyelids. Approximately 15% to 20% of patients with facial burns exhibit ocular injury. Most eyelid burns are the result of exposure to fire. In developing countries, 80% of chemical burns were due to industrial and/or occupational exposure. Approximately 60% of eyelid burns developed eyelid contractures and eyelid ectropion, leading to loss of protection of cornea [3]. The effects of corneal exposure include corneal ulcerations, corneal perforation, cataracts, glaucoma, scarring of the cornea, and ultimately loss of vision. These complications are more often caused by direct contact with chemicals. A third of patients who sustain chemical burn injuries of the eye require corneal transplants. However, the success rate of corneal transplants is less than 50%, often requiring multiple attempts before success is achieved. About 15% of eyelid burn patients can become blind if not treated promptly [3,4]. Early management is critical in the eyelid burn patient, including non-surgical measures such as the use of artificial tears and moist gauze covering of the eyes to prevent drying of the cornea. Early eyelid surgical management is critical for the protection of the cornea. Delay in surgical management may result in eyelid contractures and eyelid ectropion after grafting. We used a modified surgical method to treat eyelid burn injuries in eight patients. Our surgical procedure successfully prevented eyelid contractures, eyelid ectropion, and the need for cornea transplantation. All our patients had good eye vision and cosmetic appearance at six-month follow-up.

## Case presentation

Case 1 was a 36-year-old Chinese woman who was diagnosed with flame burn three hours after injury. Her injuries were second or third degree burns involving the total head, anterior and posterior torso, both arms and parts of both thighs. Her blood pressure was 90/60 mmHg. Liquid resuscitation and antibiotics were used upon admission. Our patient was in a stable condition after resuscitation. Eyelid surgery was performed on day three.

Case 2 was a 38-year-old Chinese man who was transferred from the emergency room (ER) 48 hours after flame burn injury. The burnt area was 80% of his body surface area (BSA), involving his head and neck, torso, both arms and parts of both legs. A total of 50% of the burn area was third degree. He was treated by oxygen inhalation, liquid resuscitation and antibiotics in the ER, and had a stable vital condition. On day three, skin graft surgery was performed.

Cases 3, 4, 5, 6 and 7 were all Chinese men aged 21, 31, 42, 44 and 55, respectively; flame burn was diagnosed three to five hours after injury with 7% to 10% BSA third degree burns involving the face, neck, both forearms and both hands.

Case 8 was a 28-year-old Chinese woman who spilled sulfuric acid on her face five hours previously. She was diagnosed as having a third degree burn on her face and second degree burns on both hands.

**Figure 1:**
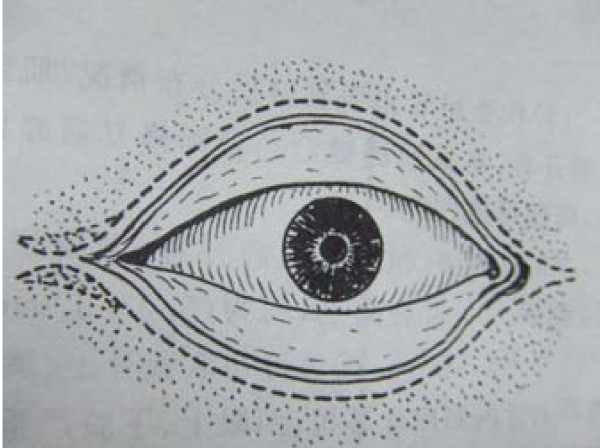
**Surgical procedures of eyelid skin graft**. Incision was made by the dashed line.

**Figure 2:**
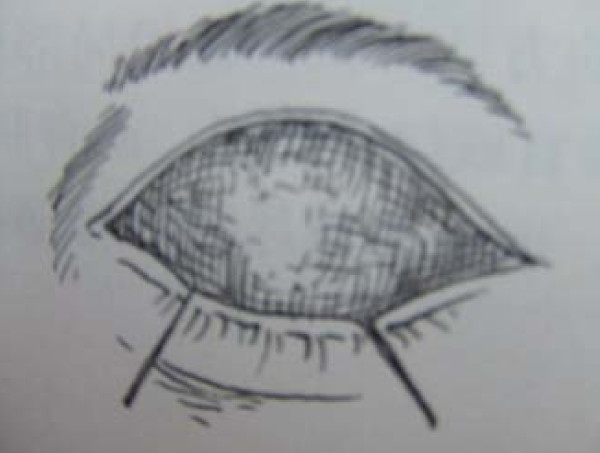
**Separation until tarsal plate with graft skin on upper lid**.

**Figure 3:**
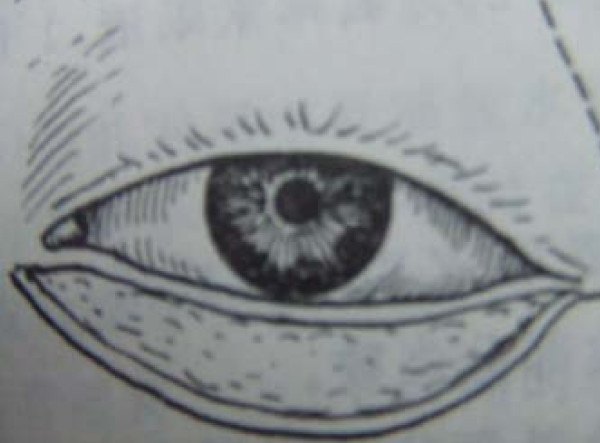
**Separation until tarsal plate with graft skin on lower lid**.

**Figure 4:**
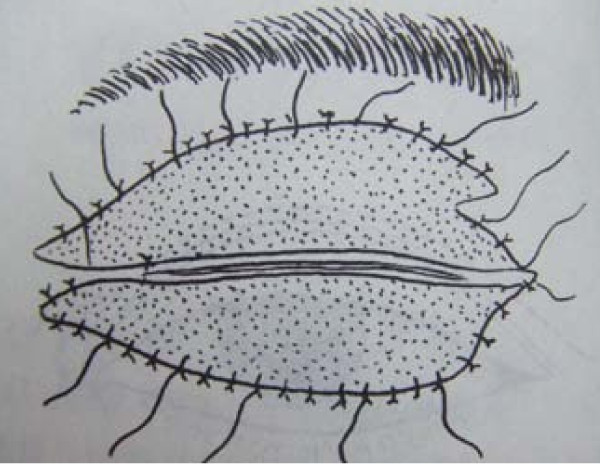
**Closed eye covered after grafting**.

**Figure 5:**
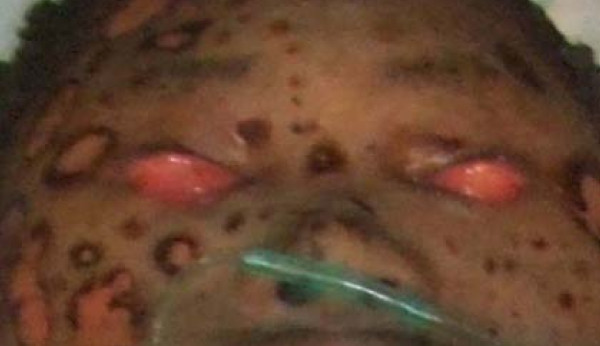
**Patient pre-operative view**.

**Figure 6:**
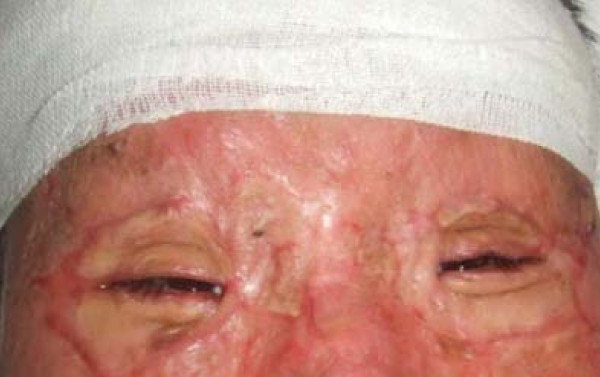
**Open eyes, seven months after surgery**.

**Figure 7:**
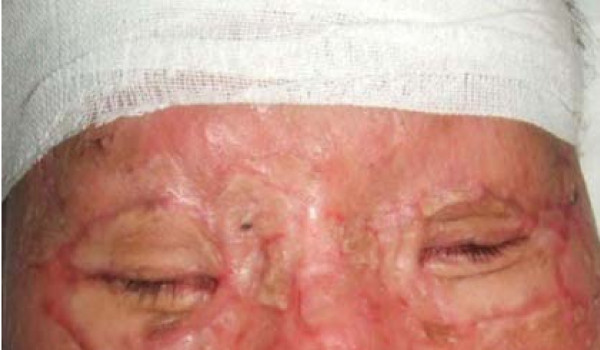
**Closed eyes, seven months after surgery**.

**Figure 8:**
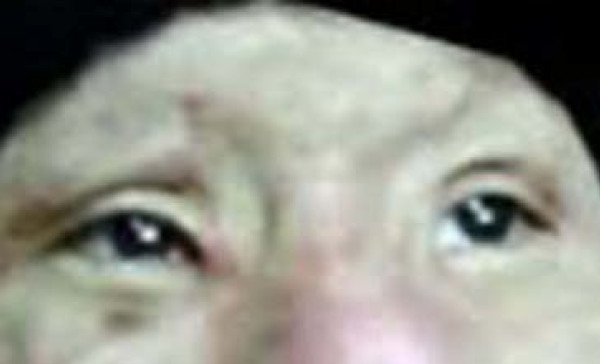
**Open eyes, 12 months after surgery**.

**Figure 9:**
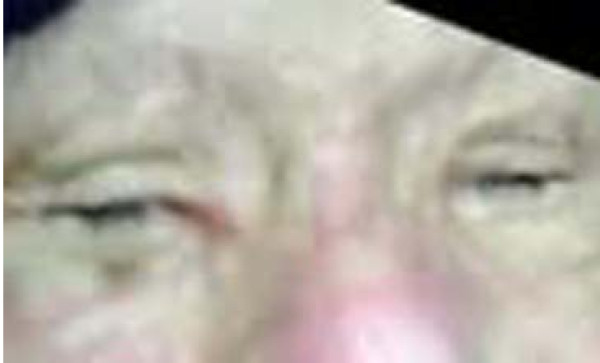
**Closed eyes, 12 months after surgery**.

All eight patients had second and/or third degree eyelid burns, and five had partial musculus orbicularis oculi burns, but with the tarsal plate intact. The patients' ages ranged from 21 to 55 years old, with a median age of 37. All our patients were hospitalized and treated with artificial tears, moist gauze eye coverings and antibiotic oculentum application to eyes twice a day for three days. Eyelid surgery was performed three days after burn injury. Surgery after three days of burn injury can avoid a large amount of plasma exudation that can influence skin graft success. Local anesthesia was induced with 1% lidocaine. A horizontal incision was made 2 to 3 mm above and parallel to the palpebral margin. The two sides of the incision passed the inner and outer oculi medialis by 5 mm. The peri-orbital areas were dermabraded. The musculus orbicularis oculi was separated and loosened between the tarsal plate and septum orbital using a fine pair of scissors. This technique can loosen the musculus orbicularis oculi, enlarging the eyelid space by 5 mm. A 15 to 20 mm wide and one-fifth longer than eyelid, full-thickness skin graft was grafted and fixed with 3-0 suture. The skin grafts were harvested from an inguinal area or thoracic area. The wound was cleaned and covered with 10% povidone-iodine gauze. Surgical sutures were removed at post-operative day 10 (Figure 1, Figure 2, Figure 3 and 4). Surgery was successfully performed on both eyes of all eight patients. The appearance, and opening and closing of eyelids were satisfactory and the protective functions were fully restored. There was no eyelid contracture and ectropion at six-month follow-up. One patient's pre-operative and post-operative follow-up pictures are included (Figure 5, Figure 6, Figure 7, Figure 8 and 9).

## Discussion

Eyelid involvement is common in facial burns. Eyelid burn injuries, especially by chemical contact, are critical emergencies. Priority is always given to eye closure, oral continence, neck and limb movement [5,6]. The acute management includes gentle eyelid and eyelash hygiene to prevent crusting. Topical ophthalmic antibiotic ointments and artificial tears should be applied frequently. The upper eyelid is responsible for moistening the cornea. Patients with eyelid burns should be examined daily, especially while asleep. When the patient is asleep the voluntary component of lid closure is lost and the cornea may be partially exposed. Tarsorrhaphy was advocated for corneal protection in the past, but it cannot prevent lid retraction in the long term. Tarsorrhaphy is not a substitute for timely skin grafting. The optimal time to perform a skin graft on an eyelid for deep second or third degree burn injury is still controversial. Most surgeons suggest grafting as early as possible. Early skin grafting increases the risks of infection and complications. However, delayed skin grafting also increases the risk of eyelid hypertrophic scarring, asymmetry and other deformities that can lead to eyelid contractures and result in cornea exposure [7-9]. We performed skin grafting at three days after burn injury. This prevented excessive exudation and contributed to skin grafting success. Our procedure also prevented large and firm scar formation and further eyelid contractures in the future [9,10]. The benefits of the new technique include a lower graft retraction rate, resulting in better corneal protection. Loosening and separating of the orbicularis muscle ensures that a big graft can be applied. This procedure makes an artificial fold on the graft, allowing extra skin for future graft contracture while still preserving the ability to close the eye adequately. We widen the grafting area by separating and loosening the musculus orbicularis oculi, gaining 5 mm of space by this technique (one-fifth longer than eyelid), which can elongate the graft by 10 mm for optimal results. We do not recommend creating more space than this, as this will cause more surgical trauma for no additional benefit. With this modified method, ectropion can be prevented even with future contraction of the wound. The use of full-thickness instead of split-thickness skin grafts, such as in our procedure, can contribute to lower ectropion incidence and lower corneal exposure [7]. The thicker the graft, the less the potential for contracture. Other advantages include increase resistance to trauma over thin grafts and less distortion functionally and cosmetically [6]. We collected these eight cases into one case series because the surgical management of the eyelid injuries was the same, although the systemic managements of these patients as different according to the injury area and type. Following our surgical procedure, our patients could close their eyes freely, with little or no eyelid retraction. The grafted eyelids also appeared more cosmetically acceptable in double folds instead of a single fold because larger skin grafts were used. Only one patient in this case series had a chemical burn injury. Thus, we need to assess the efficacy of this modified surgical procedure in more chemical burn injury patients in the future.

## Conclusions

This new technique is beneficial in the treatment of eyelid burn injuries, especially in the treatment of flame eyelid burn injury. The results for chemical burn injuries need more testing.

## Consent

Written informed consent was obtained from the patients for publication of this case report and any accompanying images. A copy of the written consent is available for review by the Editor-in-Chief of this journal.

## Competing interests

The authors declare that they have no competing interests.

## Authors' contributions

HL, KW and YJ designed the surgical procedure. QW and SD worked as team members. YJ wrote the manuscript. All authors read and approved the final manuscript.
